# Consensus recommendations on the role of nurses in the care of headache patients: protocol for a european e-delphi study

**DOI:** 10.1186/s12912-023-01211-1

**Published:** 2023-02-24

**Authors:** Annette Vangaa Rasmussen, Rigmor Hoejland Jensen, Leena Eklund Karlsson, Louise Schlosser Mose

**Affiliations:** 1 Department of Neurology, Zealand University Hospital, Roskilde, Denmark; 2 Danish Headache Center, Department of Neurology, Rigshospitalet-Glostrup, University of Copenhagen, Copenhagen, Denmark; 3 Unit for Health Promotion Research, University of Southern Denmark, Odense, Denmark; 4 Research Unit of Neurology, University hospital of Southern Denmark, Esbjerg, Finsensgade 35, 6700 Esbjerg, Denmark; 5 Department of Regional Health Research, University of Southern Denmark, Odense, Denmark

**Keywords:** Delphi-study, Expert-based treatment recommendations, Migraine treatment, Nurses treatment

## Abstract

**Background:**

Nurses play an important role in the treatment of headache patients at the specialized headache centers in Europe, however, a unified definition of nursing tasks and conduction of tasks is lacking. The objective of this e-Delphi study is twofold. Initially, to obtain healthcare professional headache experts’ opinions on which tasks are associated with nurses’ care in migraine treatment. Then, through an iterative multi-staged process, to combine the opinions into group consensus statements and develop evident European nursing recommendations for migraine treatment.

**Methods:**

In Delphi studies there are no unambiguous methodological guidelines and this protocol is being published to ensure transparency and quality in the study process. We invite nurses working in specialized headache centers and neurologists co-working with nurses in Finland, Denmark, Norway, Sweden, United Kingdom, Netherlands, Germany, Ireland, Estonia and Switzerland to participate anonymously in the expert panel. This e-Delphi study consists of three rounds of online questionnaires. We use Open-ended questions to capture the essentials of nurse tasks as understood by the expert panel members. Data are analyzed using content analysis. Predefined statements are applied for the experts to rate the importance of nurses’ tasks synthesized from a systematic examination of the existing literature. Consensus is measured using descriptive statistics; median, Interquartile range (IQR) and percentage agreement. Measurement of agreement between participants will be analyzed using inferential statistics; Kendall’s coefficient and stability between rounds; Wilcoxon rank-sum test. Statements, which receive consensus in the third round, are included in the final compilation of European recommendations for nurse care for migraine patients.

**Discussion:**

The e-Delphi study will provide European recommendations on nurse care in migraine treatment, which could not be created on the basis of the existing literature. The recommendations can open for the conduction of further research including measurement of efficacy of clinical implementation of the recommended tasks.

**Trial registration:**

The study is registered at The Region of Southern Denmark (21/52,885). According to The Regional Ethical Committee and Danish law, no additional approval is relevant (20212000-145). A written informed consent is obtained from all participants before inclusion in the study.

## Background

Migraine is a reoccurring primary headache disorder with a prevalence of 15% among the adult population in Europe [1, 2]. It is commonly known that migraine constitutes a huge financial cost for society, but migraine also has a high personal impact in the individual [3]. Migraine often affects everyday life causing social absence, decreased quality of life, lack of control, and reduced work- and functional capacity [2, 4, 5]. Consequently, many people with migraine consult general practitioners, neurologists and specialized headache centers for migraine treatment. Especially, treatment at specialized headache centers often contains both pharmacological and non-pharmacological elements including the biological, emotional and behavioral factors of migraine [6, 7]. In order to elucidate the different treatment elements, a multidisciplinary approach including e.g., neurologists and nurses are preferable [6–8]. In this study, nurses are defined from the International Council of nurses (ICN); “*The nurse is a person who has completed a program of basic, generalized nursing education and is authorized by the appropriate regulatory authority to practice nursing in his/her country”* [9].

Through the non-pharmacological treatment, the emotional and behavioral factors can be targeted, which are elements described by migraine patients as central to their management of migraine [4, 10]. These elements are an important part of the strategies for overall headache treatment. It focuses on patient-centered themes such as; *dialogue on living with a disabling disorder, the relationship between health care professionals and patients, coping with and acceptance of migraine, guidance and information, technical training for injections, evaluation of treatment efficacy and experiences of side effects, and finally treatment compliance.* All the above-mentioned themes appeal to the fundamentals in nursing, where the specific strength of nurses is to bring care to treatment. That is why these non-pharmacological elements mostly are performed as part of nurses’ tasks. Despite the sparse evidence for non-pharmacological treatment tasks, many nurses in Europe provide these tasks on a daily basis. There is a lack of evidence and consensus on which tasks to be conducted and how nurses working in headache clinics and at specialized headache centers in Europe could do these. Generally, there is not a special education for headache nurses and it varies from country to country in Europe, how the nurse acquires knowledge to give care to headache patients, why evidence based recommendations can be an advantage in their daily work.

Looking at the existing literature, the nurses’ role in migraine treatment is described sparsely. Differences in study perspectives, descriptions of tasks, and variations in study designs make comparisons difficult. Therefore, customizing the nursing tasks across organizations and settings based on existing literature is challenging. Previous studies have primarily focused on nurses working in primary care settings and these studies have been national orientated and not focused on development and unification of nursing across countries. The topics include e.g., nurse-led education for empowering patients to manage migraine [11], nurse administered screening for diagnosis of migraine [12, 13], supervised nurse consultations [14, 15], and costs and benefits of migraine nurses [16]. Existing literature and experiences from international conferences for headache nurses indicate that nurse tasks could use transparency e.g. by the development of evidence-based recommendations for nurse care in migraine treatment. For optimal headache care of patients, nurses must work confidentially and professionally, which could be achieved by consulting evidence-based recommendations. Based on the abovementioned, the present study shall result in European recommendations for the nurse’s care in the treatment of migraine patients.

The aim of this e-Delphi study is twofold. Initially, to obtain healthcare professional headache experts’ opinions on which tasks are associated with nursing in migraine treatment. Then, through an iterative multi-staged process, the researchers will combine the opinions into group consensus statements on which nursing-specific tasks should be included in the European nurse recommendations for migraine treatment and on how these tasks should be managed.

## Methods

The Delphi method is applicable for generating insights especially in situations with limited availability of information. It is a systematic approach to obtaining a consensus among experts [17]. Consensus is achieved by the completion of sequential questionnaires which are redefined by feedback and resulting in a convergence of opinions and probable consensus [17]. The Delphi method is advantageous due to iteration with controlled feedback and the possibility of amalgamation of information from experts. Moreover, the anonymous participation is valuable, as it reduces potential halo effects, where dominant group members are giving extra credence and subject to bias [18, 19]. As two different health care professions are included, there is a potential be variation in which tasks, the nurses should perform and it is essential that all statements are equally rated. Furthermore, the use of electronic questionnaires in an e-Delphi design remove all geographical limitations [17], which could otherwise occur, since this is a European study including participants from different countries.

### Study design

**Fig. 1:**
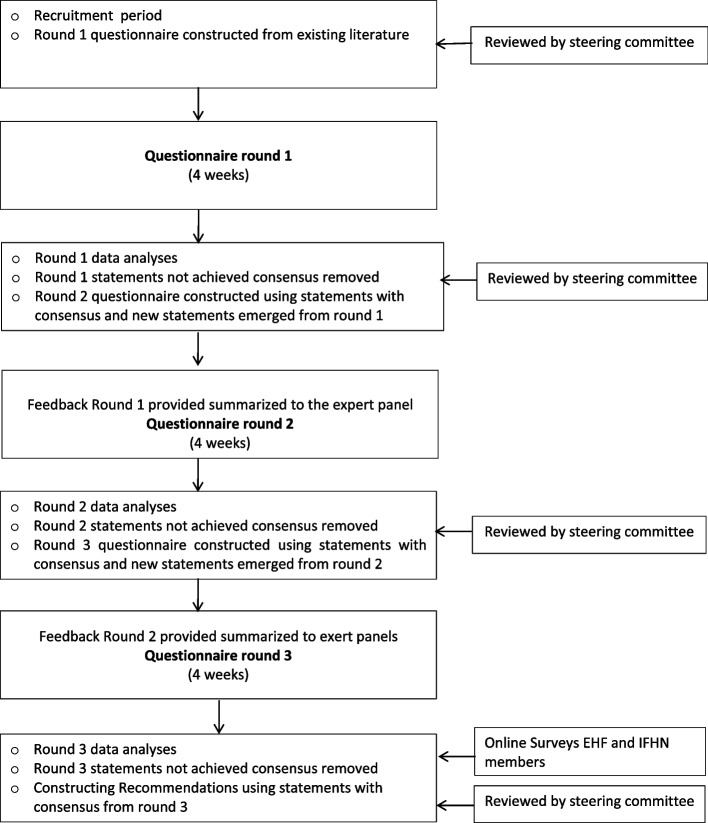
Flow of the three e-Delphi questionnaire rounds

This e-Delphi study is conducted with inspiration from the Guidance on Conducting and Reporting Delphi Studies (CREDES) and supplemented by recommended criteria for Delphi studies [20, 21]. Delphi studies have no unambiguous methodological guidelines and this protocol is being published to ensure transparency and quality in the study process. The study consists of three rounds of questionnaires among an expert panel combined with an online survey inviting all European Headache Federation members (EHF) and members from the International Forum for Headache Nurses (IFHN) to comment and give feedback before conducting the final European recommendations. The flow through the e-Delphi study is illustrated in Fig. 1.

A combination of open and closed questions will guide the questionnaire for the first round [22]. Open-ended questions will capture the essentials in nurse tasks as understood by the expert panel members. In closed questions, the headache experts will rate the importance of specific nurses’ tasks. In the two subsequent rounds of questionnaires, the experts will rate their level of agreement with statements about nurse tasks using a 5-point Likert-scale. As part of the process, the expert panel is informed about the responses from the previous round of questionnaire in a summarized form in the next questionnaire round. This allows the respondents to reconsider their replies and potentially revise them in the next round [22, 23]. Therefore, the Delphi design is an iterative multi-staged process with the purpose to combine opinions into a group consensus [24, 25]. Existing literature confirms that three rounds of questionnaires are sufficient for obtaining consensus [26, 27]. Tasks and statements, which receive consensus in the third round, will be included in the final compilation of recommendations for nurse care in migraine treatment.

### Eligibility and sample for the expert panel

There are no unified guidelines, which determine sample size in Delphi studies, as it depends on the purpose of the study, the selected design and period of data collection [17, 21]. Previous studies, which aimed to define working tasks and elements in treatment, typically reached consensus with response from 10 to 17 experts in the final round [28, 29]. One neurologist and two nurses from specialized headache centers in each of the ten countries are invited. This to ensure a predominance of nurses with approximately a 1:2 distribution in the expert panel.

A purposive sampling technique is used [30]. Therefore, the participants are not selected randomly and the representativeness cannot be assured. The inclusion of a heterogeneous sample will ensure, that a variety of opinions are determined [31, 32]. To ensure a broad spectrum of opinions and statements, experts are defined as either nurses working at specialized headache centers or neurologists co-working with nurses in specialized headache centers:

- Nurses working in specialized headache centers: Eligible nurses have ≥ 2 years of experience working with migraine patients, work at a specialized headache center, are able to read and write English, and are willing to participate.
- Neurologists co-working with nurses at specialized headache centers: Eligible neurologists have ≥ 2 years of experience in co-working with nurses at a headache center, are able to read and write English and are willing to participate.

Experts from specialized headache centers in Finland, Denmark, Norway, Sweden, United Kingdom, Netherlands, Germany, Ireland, Estonia and Switzerland are invited by email to participate. Potential experts are located through the fora of European Headache Federation (EHF) and the International Forum for headache Nurses (IFHN). Upon confirmation of interest and eligibility, the experts receive an information sheet and a link to RedCap, which is a secure web application for building and managing of online surveys [33]. Prior to enrollment in the study, the participants must fulfill the electronic consent form in RedCap.

### Procedures

Data are collected between May 2022- September 2023. The questionnaire in each round is completed electronically and stored anonymously in RedCap. The expert panel has four weeks to respond to the questionnaires. E-mail reminders are sent twice to non-responders within the four week-period. Estimated time consumption for each of the questionnaire is 20–30 min. The participants can save the questionnaires at any time and return later.

### Round one

Prior to developing the questionnaires, a systematic literature search is carried out on databases; PubMed, CINAHL, Web of Science and EMBASE for studies about nursing in migraine treatment. This search is supplemented by publications identified through google scholar. The steering committee reads and discuss the studies and afterwards the studies are grouped into four overall width themes based on the nursing tasks described: 1. Screening and anamnesis, 2.Communication, 3. Medication, 4. Education and information of patients.

The first round questionnaire is divided into three sections. In the first section, demographic data of the expert panel members is collected. In the second section, experts rate the level of importance of the predefined statements regarding nurse tasks in migraine treatment as per the above mentioned literature study. Therefore, this section includes statements elicited from the existing studies on nurse tasks such as; “*Nurses give guidance to patients on how to complete a headache diary”* or *“Nurses inform patients about use of acute medical treatment”.* The level of importance of each statement will be rated using a *5-point Likert-scale from 1 = Not important at all – 5 = Very important.* The third section of the questionnaire consists of the open- ended question; *“*In your own words, which tasks might be better performed (more efficiently, more cost-effectively and/or with expectation of better outcomes) by nurses in the care of migraine patients??” This question will be probed to get as many perspectives and ideas to nurses tasks as possible in the first round. The first round questionnaire is tested for relevance, readability, accessibility and user-friendlily within the steering committee and adjusted accordingly.

### Round two

In the second round, statistics and themes are summarized and a new nuanced questionnaire is prepared based on the answers and results from the first round. The questionnaire in this round is designed to elaborate the tasks to be included in the final recommendations. Again, in the 2nd round, the questionnaire contains statements to rate and open-ended questions. Statements which achieve consensus in round one are included and the expert panel is asked to rate their level of agreement of each statement using the same 5-point Likert-scale. After each statement there is space for additional comments. At the end of the questionnaire, open-ended questions give the experts the opportunity to reflect and state opinions about the tasks and conduction.

### Round three

The third round questionnaire presents a summary from the previous round using descriptive statistics and themes emerged from the open-ended questions. By this, the expert panel can reflect before completing the last questionnaire part in the study. In the third round only statements, achieving consensus in round two, are included. Other previous statements are discharged due to lack of consensus in the second round. The panel will rate their level of agreement with the included statements using a 5-point Likert scale and the respondents can clarify their responses in a and a text. New themes will not be included at this point.

After completion of the third round questionnaire data are analyzed and a preliminary version of the final recommendations is presented in an online survey inviting feedback through the EHF and IFHN websites. This survey is conducted to reach out to as many nurses and neurologists from the European headache centers and clinics as possible for a broader input for the final recommendations. The principal investigator and the secondary investigator will conduct analyses of the responses from the online survey and the steering committee will review and discuss the findings. If new themes emerge from the feedback from the online survey further rounds of the e-Delphi process could be necessary.

### Data analyses

Qualitative data is proceeded using QSR international’s NVivo V12 and the principal investigator and the secondary investigator conduct analyses between each round. For the qualitative data the investigators conduct content analysis inspired by the analytic steps of Graneheim and Lundman [34]. A content analysis is applicable for analyzing written or verbal communication in a systematic way [35]. This analytic approach is sufficient to identify content areas and to combine or collapse similar statements from the expert panel into new statements for the next questionnaire round [17, 34]. Initially, the answers to open-ended questions are read several times. Thereafter, the analyses is conducted through an iterative process with several steps; (1) Detection of content areas emerged with little interpretation of data, (2) Division of content areas to meaning units based on related content and context, (3) Coding meaning units, (4) Interpretation of codes based on similarities and differences and division of codes to tentative categories to, be formulated into themes [34, 36], which can be proceeded and elaborated in the next questionnaire round.

Between each questionnaire round, the steering committee reviews and discuss the summarized data for feedback and the principal investigator edit accordantly before dissemination.

Quantitative data are analyzed in Stata17 and evaluated within three areas; Consensus, agreement and stability using descriptive and inferential statistics. The pre-defined level of consensus, stability and agreement is used to evaluate the inclusion of statements in the next round questionnaires. Consensus is defined as the extent to which the expert panel members share the same opinion [17]. As the Likert scale is considered an ordinal scale [37] consensus will be statistically summarized for central tendencies and dispersion using a prior criteria for median and interquartile range (IQR). Furthermore, for each statement percentage of agreement with responses rated *important/very important* are also used to evaluate consensus. Statements which fail to reach consensus according to a prior defined criteria are discarded before the next questionnaire round. In Table 1 the criteria for statistical measurements of consensus, agreement and stability in each round are lined out. Kendall’s coefficient of concordance with 0 being no agreement and 1 being perfect agreement is used to evaluate agreement between experts across statements. Stability of responses between questionnaire rounds will be analyzed using the Wilcoxon rank-sum test. *P*-values of *p* < 0.05 is considered statistical significant in all analyses.

**Table 1:** Criteria for statistical measurements of consensus, agreement and stability

	Statistics	Round 1	Round 2	Round 3
**Consensus**	Median Interquartile range (IQR) Percentage agreement	≥ 3 ≤ 1.5 ≥ 60%	≥ 3 ≤ 1.5 ≥ 65%	≥ 3.5 ≤ 1 ≥ 70%
**Agreement**	Kendall’s coefficient of concordance (*W*)	*P* < 0.05	*P* < 0.05	*P* < 0.05
**Stability**	Wilcoxon rank-sum test	NA	*P* < 0.05	*P* < 0.05

### Ethics

The study is registered at The Region of Southern Denmark (21/52,885). According to The Regional Ethical Committee and Danish law, no additional ethical approval is relevant (20212000-145). Written study information is given to participants prior to inclusion. A written informed consent is obtained from all participants prior to inclusion in the study and answering questionnaires. All participants are informed about the possibility of withdrawing from the study at any time. Confidentiality is secured, as personal information will be collected anonymously and stored in RedCap. Only anonymized data are shared within the steering committee of this study and the participants will remain anonymous to the other participants and in any written reports.

## Discussion

From existing literature, it is known that migraine patients find treatment of non-pharmacological elements such as emotional and behavioral factors important for management [4, 10]. Especially, this treatment approach appeals to the strength and fundamental in nursing. However, it is still unclear how to unify and make visible the role of nursing. This e-Delphi study will provide European consensus on the nursing tasks in migraine treatment, which could not be established on the basic of the existing literature.

The e-Delphi design allows for development of expert-based recommendations within the nursing field of migraine. Anonymous participation is considered a strength, as this prevent a halo effect where dominant group members are giving extra credence [19]. The selection of experts can be a challenge and potentially create selection bias. However, no established guidelines exist on inclusion strategies and numbers to be included and it highly depends on the research field [17, 26]. In this study, we will include a variety of experts from predefined eligibility criteria to ensure a variety of experts with good clinical experience, different background and nationality.

Statements in the questionnaire rounds are constructed from expert opinions and existing research, reflecting both clinical practice and known evidence. The use of statements based on existing literature in the first round questionnaire can potentially induce bias. We believe, however, that the combination of both fixed statements and open-ended questions will allow for inspiration and reflection on the tasks from a clinical perspective. There is no guidance in relation to balance the collection of both qualitative and quantitative data, even though this is often used in Delphi-studies [17].

The steering committee discussed the relevance of including patient representatives as key stakeholders. As little is known about the tasks and nurses’ role prior to this study, we decided to include neurologists and nurses. The conduction of this study will open for studies evaluating the implementation of the nurse recommendations as well as investigating the patients’ perspectives on nurses’ care in the clinical settings.

## Clinical implications

This study emerged from clinical work with migraine patients and an obvious need to create a common foundation for nurses’ tasks in treatment. All the participants in this study contribute to the development of European clinical nurse recommendations for migraine treatment. From the recommendations, it is possible to conduct further research including measurement of efficacy from clinical implementation of the recommended tasks. Clinically, the development of recommendations will have a positive impact on the unification of tasks, and further development of nurse care for the benefit of the migraine patients. This e-Delphi study focus on nurses care to migraine patient, however many of the nurses tasks probably could be used in a broader context in general headache care.

## Data Availability

The datasets generated during and/or analysed during the current study will be made available from the corresponding author on reasonable request.

## Abbreviations

EHF: European Headache Federation
IFHN: International Forum for Headache Nurses
IQR: Interquartile range
